# The Psychological Impact of COVID-19 Pandemic on Health Care Workers: A Systematic Review and Meta-Analysis

**DOI:** 10.3389/fpsyg.2021.626547

**Published:** 2021-07-08

**Authors:** Ping Sun, Manli Wang, Tingting Song, Yan Wu, Jinglu Luo, Lili Chen, Lei Yan

**Affiliations:** ^1^Center for Reproductive Medicine, Cheeloo College of Medicine, Shandong University, Jinan, China; ^2^The Eighth People's Hospital of Xinjiang Uygur Autonomous Region, Urumqi, China; ^3^Chen Lili's Clinic, Korla, China

**Keywords:** mental health, anxiety, depression, insomnia, COVID-19, health care workers

## Abstract

**Objective:** The COVID-19 epidemic has generated great stress throughout healthcare workers (HCWs). The situation of HCWs should be fully and timely understood. The aim of this meta-analysis is to determine the psychological impact of COVID-19 pandemic on health care workers.

**Method:** We searched the original literatures published from 1 Nov 2019 to 20 Sep 2020 in electronic databases of PUBMED, EMBASE and WEB OF SCIENCE. Forty-seven studies were included in the meta-analysis with a combined total of 81,277 participants.

**Results:** The pooled prevalence of anxiety is 37% (95% CI 0.31–0.42, I^2^ = 99.9%) from 44 studies. Depression is estimated in 39 studies, and the pooled prevalence of depression is 36% (95% CI 0.31–0.41, I^2^ = 99.6%). There are 10 studies reported the prevalence of insomnia, and the overall prevalence of insomnia is 32% (95% CI 0.23–0.42, I^2^ = 99.5%). The subgroup analysis showed a higher incidence of anxiety and depression among women and the frontline HCWs compared to men and non-frontline HCWs respectively.

**Conclusions:** The COVID-19 pandemic has caused heavy psychological impact among healthcare professionals especially women and frontline workers. Timely psychological counseling and intervention ought to be implemented for HCWs in order to alleviate their anxiety and improve their general mental health.

## Introduction

Epidemic studies proved that previous infectious diseases caused long-term and persistent psychopathological consequences among this category. Similarly, in this extremely hard and bitter fight against COVID-19, health care workers (HCWs) played a significant role and may undergo severe psychological stress. Just like several studies reported, a multitude of HCWs appeared to suffer from several long-lasting psychological problems, including anxiety, depression, insomnia, etc. (Cheng et al., 2020). HCWs are exposed to longer work shifts in order to meet the growth of health care demand. Meanwhile, the lack of social support, poor sleep quality, isolation from families and friends, fear of spreading the disease to their families and coworker and direct contact with patients are several triggers for more psychological problems among HCWs. Moreover, during working hours, HCWs have to wear protective equipment making their movement and operation slowly and cause respiratory discomfort and difficulties which are also aggravating factors for psychological symptoms of HCWs (Cheng et al., 2020; Elhadi et al., 2020; Giusti et al., 2020; Huang et al., 2020a).

A systematic review and meta-analysis summarizes COVID-19 during a pandemic the prevalence of depression and anxiety among the healthcare workers, which includes only the literatures published in Asia before April 2020 (Luo et al., 2020). Following the publication of this review, many studies have been published on the psychological impact of COVID-19 on health professionals in many other countries and have compared the psychological impact of COVID-19 on frontline and non-frontline HCWs. As the epidemic continues to spread and cases increase at this stage, we consider that a meta-analysis of published studies is necessary to explore whether COVID-19 has further psychological effects on medical staffs. In order to implement appropriate strategies to prevent or intervene in the adverse psychological effects on HCWs and provide help to relieve the burden, we conducted the current systematic review and meta-analysis to assess the latest psychological impact of the COVID-19 pandemic among healthcare workers In this meta-analysis, we assessed the psychological impact of COVID-19 on the HCWs and summarized the different prevalence rates of anxiety and depression between frontline HCWs and non-frontline HCWs.

## Methods

### Literature Search

In order to perform a systematic review and meta-analysis on studies evaluating the prevalence of the psychological and mental impact of COVID-19, we did this study according to the Preferred Reporting Items for Systematic Reviews and Meta-Analyses (PRISMA) statement (Liberati et al., 2009). The study was approved by the Ethics Committee of the Eighth People's Hospital of Xinjiang Uygur Autonomous Region. We searched the original literatures published from 1 Nov 2019 to 20 Sep 2020 in electronic databases of PUBMED, EMBASE and WEB OF SCIENCE. Our search terms were (“COVID-19”/exp OR COVID-19 OR “coronavirus”/exp OR coronavirus) AND (“psychological”/exp OR psychological OR “mental”/exp OR mental OR “stress”/exp OR stress OR “anxiety” OR anxiety OR “depression” OR depression OR “post-traumatic” OR “post-traumatic”/exp OR “trauma” OR 'trauma'/exp) AND (“doctor” OR “maternity staff” OR “medical staff” OR “medical workers” OR “Healthcare workers” OR “Healthcare staff” OR “Healthcare professional” OR “Nursing professional” OR “nursing staff” OR “nurses”) for EMBASE; [(“COVID-19”[All Fields] OR “coronavirus”[All Fields]) AND (“Stress, Psychological”[Mesh] OR “mental” OR “anxiety” OR “depression” OR “stress” OR “post-traumatic” OR “trauma”)] AND [[[[[[[[[(doctor) OR (maternity staff)] OR (medical staff)] OR (medical workers)] OR (Healthcare workers)] OR (Healthcare staff)] OR (Healthcare professional)] OR (Nursing professional)] OR (nursing staff)] OR (nurses)] for PUBMED; #1 TS=(COVID-19 OR coronavirus), #2 TS = (Psychological OR mental OR anxiety OR depression OR stress OR post-traumatic OR trauma), #3 TS=(doctor OR surgeons OR surgical staff OR maternity staff OR medical staff OR medical workers OR healthcare workers OR healthcare staff OR healthcare professional OR nursing professional OR nursing staff OR nurses), #4 #1 AND #2 AND #3 for WEB OF SCIENCE.

### Selection Criteria

We selected the literatures we need according to our PICOS (population; intervention; compare; outcomes; study) criteria. The included criteria of our study were as follows: (1) P/I: The subjects in these literatures should be healthcare staffs (i.e., medical doctors, nurses, nursing assistant, clinical assistant departments' staffs, public health professionals) fighting against the COVID-19; (2) O: Only when those studies evaluating the prevalence rates of anxiety, depression and/or insomnia using validated assessment methods were eligible for inclusion; (3) S: Cross-sectional studies, cohort studies, randomized controlled trials were acceptable; (4) The language of the included studies is English or Chinese.

Two researchers searched the literatures independently, and we conducted three rounds of screening of the studies we have searched. Firstly, the titles of the articles were screened and then the selected studies are further screened by reading abstracts of the literature. Finally, the full text of the articles after the second round of screening were read in order to decide which studies would be included. If two researchers had discrepancies on whether to include a certain study, the senior author (LY) was consulted to make the final decision.

### Data Extraction and Quality Assessment

The information from each literature were extracted by two researchers independently including: author, year, the number of the participation, response rate, region, the percentage of physician, nurse and other healthcare workers in those studies, the percentage of men and women in included articles, survey methods used and the cut-offs mentioned in each study as well as the total number and percentage of participants that screened positive for depression, anxiety or insomnia. As far as the quality assessment method, we referred to a resent systemic review and meta-analysis (Pappa et al., 2020) using the modified Newcastle-Ottawa scale to evaluate the quality of included cross-sectional studies. The appraisal tool assessed the representativeness of sample and the sample size, the validate assessment tool with appropriate cut-offs, the response rate and adequacy of descriptive statistics. The total score ranged between 0 and 5. Studies scoring ≥3 points were regarded as low risk of bias, compared to the studies assessed with <3 points that were regarded as high risk of bias.

### Data Synthesis and Analysis

The metan and metaprop module in STATA was used to calculate the pooled prevalence and 95% confidence interval of anxiety, depression and insomnia with random effects models (Luo et al., 2020). Substantial heterogeneity was defined as I^2^ > 75%. We also perform a subgroup analysis according to the following categories: the severity of anxiety and depression, gender, frontline and non-frontline healthcare workers. In articles that focus on the severity of anxiety and depression, the following questionnaires and grading criteria are used. Generalized Anxiety Disorder 7 (GAD-7) questionnaire:mild (score of 5–9), moderate (score 10–14), or severe (score 15–21) (Spitzer et al., 2006); the Patient Health Questionnaire (PHQ-9):mild (score of 5–9), moderate (score 10–14), moderately severe (score 15–19), or severe (score 20–27) (Löwe et al., 2004); Selfrating Anxiety Scale (SAS): a score of 50–59 indicated mild anxiety, 60–69 indicated moderate anxiety, and ≥70 indicated severe anxiety; Self-Rating Depression Scale (SDS): 50–59 for mild depression, 60–69 for moderate depression, and 70 or more for severe depression (Zung, 1971); The Hamilton rating scale for anxiety (HAMA): no anxiety (score 0–6), mild and moderate anxiety (score 7–13), severe anxiety (score ≥ 14); The Hamilton rating scale for depression (HAMD): mild and moderate (score 7–23), severe depression (score ≥ 24) (Lu et al., 2020). Frontline HCWs are defined as those who currently caring for COVID-19 patients in the participating hospitals and working in places with the highest probability of contact with COVID-19, for example, intensive care units, infectious diseases units, and emergency departments (Hu et al., 2020; Rossi et al., 2020; Wańkowicz et al., 2020). The non-frontline HCWs are those not working with COVID-19 patients (Lai et al., 2020). Sensitivity analysis was done by subtracting each study and calculating the pooled prevalence of the remaining studies, in order to identify studies which may severely affect the pooled prevalence. Our main outcomes were prevalence (p), confidence intervals (CI) and percentage prevalence (*p* × 100%).

## Results

### Search Result and Characteristics of Studies

**Figure 1:**
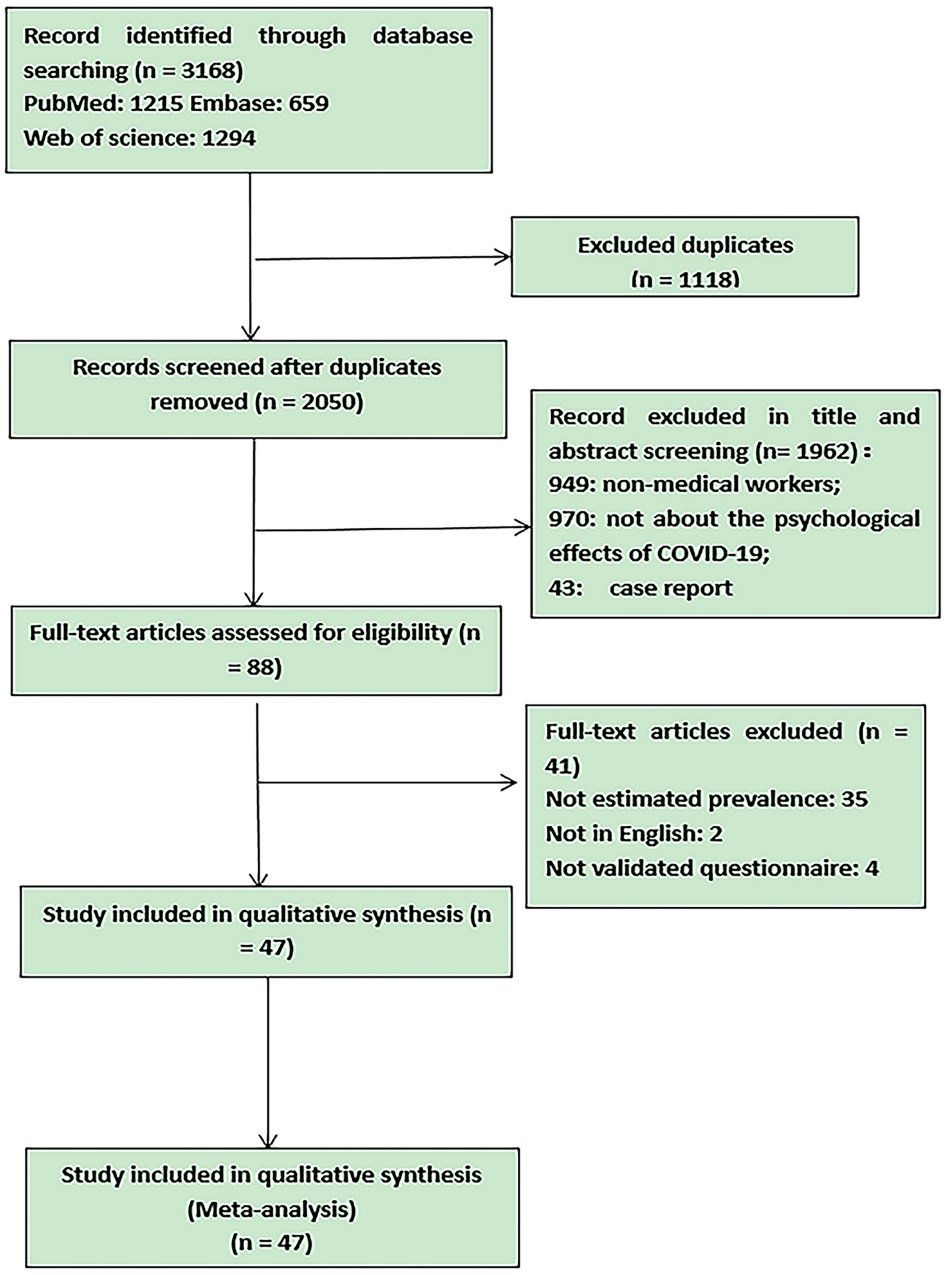
Flow chart of literature screening.

A PRISMA diagram detailing the study retrieval process is shown in Figure 1.

The total number of the references we had searched from three databases was 3,168 (Embase: *n* = 659; PubMed: *n* = 1,215; Web of science: *n* = 1,294). Among these articles, 1,118 studies were removed because of duplication. After screening the title and abstract, 1,962 studies were excluded due to failure to meet the inclusion criteria (949: non-medical workers; 970: not about the psychological effects of COVID-19; 43: case report). Eighty-eight studies were screened for the full text. Among the above studies, 35 were deleted because of not reporting the prevalence, four were excluded because no mention was made of the type of questionnaire, and two were deleted because not in English or Chinese. Finally, 47 studies are included in this meta-analysis. All of these studies are cross-sectional studies and they are all conducted through online questionnaires (“questionnaire star,” Wechat or text message/email, etc.). A trigraph has been made to show the characteristics of these studies in Table 1. The results of the quality assessment for each study using Newcastle-Ottawa score are presented in Table 2, and the total pooled prevalence of anxiety, depression and/or insomnia as well as the subgroup analysis results are showed in the figures below.

**Table 1:** The characteristics of included studies.

**References**	**Study population**	**Response rate (%)**	**Region**	**Age (MD ± SD/RANGE)**	**Physicians (%)**	**Nurses (%)**	**Others (%)**	**Female (%)**	**Assessment**	**Cut off**	**Depression (%)**	**Anxiety (%)**	**Insomnia (%)**
Hosseinzadeh-Shanjani et al. (2020)	200	100%	Iran	40.6 ± 10.15	21.50%	47%	31.50%	80%	DASS	N.A.	46 (23%)	40 (20%)	N.A.
Liu et al. (2020)	512	85.40%	China	18–39	N.A.	N.A.	N.A.	84.50%	SAS	≥50	N.A.	64 (12.5%)	N.A.
Guo et al. (2020)	11,118	N.A.	China	20–50	30.40%	53.07%	16.53%	74.80%	SAS	≥50	3858 (13.47%)	554 (4.98%)	N.A.
									SDS	≥50			
Tan et al. (2020)	470	94.00%	Singapore	34.4 ± 5.85	28.70%	34.30%	37%	68.3	DASS-21	D>9	42 (8.9%)	68 (14.5%)	N.A.
										A>7			
Wang et al. (2020)	1,045	99.60%	China	-	N.A.	N.A.	N.A.	N.A.	HADS	≥7	411 (39.4%)	142 (13.6%)	521 (49.9%)
									ISI	≥7			
An et al. (2020)	1,103	100%	China	32.2 ± 7.61	-	100%	-	90.80%	PHQ-9	≥5	43.60%	N.A.	N.A.
Azoulay et al. (2020a)	1,058	67%	France	27–42	29.10%	68%	2.60%	71%	HADS	≥7	30.40%	50.40%	N.A.
Chen X. et al. (2020)	252	62.80%	Ecuador	39–53	N.A.	N.A	N.A.	65.50%	GAD-7	≥10	N.A.	71 (28.2%)	N.A.
Cheng et al. (2020)	534	N.A.	China	-	54.10%	45.90%	-	82.40%	SAS	≥50	N.A.	14.00%	N.A.
Elhadi et al. (2020)	745	93.10%	Libyan	33.7 ± 7.4	N.A.	N.A.	N.A.	51.90%	HADS	≥11	420 (56.3%)	348 (46.7%)	N.A.
Giusti et al. (2020)	235	71.20%	Italy	44.6 ± 13.5	42.20%	26.00%	31.80%	62.60%	DASS	D>9	26.80%	31.30%	N.A.
										A>7			
Hu et al. (2020)	2,014	77.50%	China	30.99 ± 6.17	N.A.	100%	N.A.	87.10%	SAS	≥50	41.40%	43.60%	N.A.
									SDS	≥50			
Huang et al. (2020a)	230	93.50%	Fu yang	32.6 ± 6.20	30.40%	69.60%	-	81%	SAS	≥50	N.A.	53 (23.04%)	N.A.
Huang et al. (2020b)	364	96.60%	Sichuan	30–40	-	32.70%	67.30%	58.50%	SAS	≥50	N.A.	85 (23.4%)	N.A.
Huang and Zhao (2020)	2,250	85.30%	China	-	N.A.	N.A.	N.A.	54.60%	GAD-7	≥9	446 (19.8%)	802 (19.8%)	531 (23.6%)
									CES-D	≥28			
									PSQI	≥7			
Labrague and De Los Santos (2020)	325	N.A.	Philippines	30.94 ± 6.67	-	100%	-	74.80%	The COVID-19	≥9	N.A.	123 (37.8%)	N.A.
									Anxiety Scale				
Lai et al. (2020)	1,257	68.70%	Wuhan	26–40	39.20%	60.80%	-	23.30%	GAD-7	≥5	634 (50.4%)	560 (44.6%)	427 (34%)
									PHQ-9	≥ 5			
									ISI	≥8			
Li et al. (2020a)	4,369	82.20%	Wuhan	30–49	46.40%	40.00%	26.80%	100%	GAD-7	≥8	620 (14.2%)	1100 (25.2%)	N.A.
									PHQ-9	≥10			
Li et al. (2020b)	176	100%	Wuhan	20–40	-	100%	-	77.30%	HAMA	≥7	N.A.	136 (77.3%)	N.A.
Li et al. (2020c)	948	N.A.	China	20–40	N.A.	N.A.	N.A.	86.82%	AIS	≥6	N.A.	N.A.	311 (32.8%)
Liu et al. (2020)	4,679	N.A.	China	18–39	39.60%	60.40%	-	82.30%	SAS	≥50	1619(34.6%)	749(16.0%)	N.A.
									SDS	≥50			
Lu et al. (2020)	2,299	94.90%	China	35.9 ± 9.0	88.80%	-	11.20%	77.60%	HAMA	≥7	269(11.7%)	568(24.7%)	N.A.
									HAMD	≥7			
Naser et al. (2020)	1,163	N.A.	Jordan	18–29	48.20%	13%	38.80%	56.10%	GAD-7	≥5	277 (23.8%)	152 (13.1%)	N.A.
									PHQ-9	≥5			
Ni et al. (2020)	214	61.20%	China	-	-	-	-	N.A.	GAD-2	≥3	41(19.2%)	47(22%)	N.A.
									PHQ-2	≥3			
Pouralizadeh et al. (2020)	441	N.A.	Iran	36.34 ± 8.74	-	100%	-	95.20%	GAD-7	≥10	313 (71%)	324 (73.5%)	N.A.
									PHQ-9	≥10			
Que et al. (2020)	2,285	N.A.	China	31.06 ± 6.99	37.64%	9.10%	53.26%	69.06%	GAD-7	≥ 5	1,013 (44.37%)	1,052 (46.04%)	657 (28.75%)
									PHQ-9	≥ 5			
									ISI	≥8			
Rossi et al. (2020)	1,379	N.A.	Italy	-	31.40%	34.23%%	34.37%	77.20%	GAD-7	≥15	341 (24.73%)	273 (19.8%)	114 (8.27%)
									PHQ-9	≥15			
									ISI	≥22			
Sandesh et al. (2020)	112	N.A.	Pakistan	-	N.A.	N.A.	N.A.	42.90%	DASS	N.A.	81 (72.3%)	96 (85.7%)	N.A.
Si et al. (2020)	863	76.00%	China	-	43.70%	24.20%	31.90%	70.70%	DASS-21	N.A.	117 (13.6%)	120 (13.9%)	N.A.
Song et al. (2020)	14,825	N.A.	China	34 ± 8.2	41.60%	58.90%	-	64.30%	CES-D	≥16	3,736 (25.2%)	N.A.	N.A.
Tu et al. (2020)	100	100%	Wuhan	34.4 ± 5.85	-	100%	-	100%	GAD-7	≥4	46 (46%)	40 (40%)	N.A.
									PHQ-9	≥4			
Wańkowicz et al. (2020)	441	N.A.	Poland	40.47 ± 4.93	N.A.	N.A.	N.A.	52.20%	GAD-7	≥4	312 (70.7%)	284 (64.4%)	N.A.
									PHQ-9	≥4			
									ISI	≥8			
Xiao et al. (2020)	705	73.60%	Wuhan	-	39.50%	37.50%	23%	67.20%	HAD	≥4	409 (58%)	382 (54.2%)	N.A.
Xiaoming et al. (2020)	8,817	N.A.	Chongqing	-	36.40%	53.10%	10.40%	78%	GAD-7	≥5	2,821 (30.2%)	2,381 (20.7%)	N.A.
									PHQ-9	≥5			
Zhang et al. (2020a)	1,563	N.A.	China	25–40	29.00%	62.90%	7.90%	82.70%	GAD-7	≥5	792 (50.7%)	699 (44.7%)	564 (36.1%)
									PHQ-9	≥5			
									ISI	≥8			
Zhang et al. (2020b)	927	N.A.	China	-	73.40%	26.60%	-	N.A.	GAD-2	≥3	811 (87.8%)	806 (87%)	571 (61.6%)
									PHQ-2	≥3			
									ISI	≥8			
Zhao et al. (2020)	972	N.A.	Wenzhou	34.16 ± 8.06	N.A.	N.A.	NA.	N.A.	GAD-7	N.A.	313 (32.2%)	438 (45.1%)	380 (39.1%)
									PHQ-9				
									ISI				
Zhu et al. (2020a)	165	100%	Gansu	-	47.90%	52.10%	-	83%	SAS	≥50	73 (44.2%)	33 (20%)	N.A.
									SDS	≥50			
Zhu et al. (2020b)	5,062	77.10%	Wuhan	30–49	19.80%	67.50%	12.70%	85.00%	GAD-7	≥8	683 (13.5%)	1,220 (24.1%)	N.A.
									PHQ-9	≥10			
AlAteeq et al. (2020)	502	N.A.	Saudi Arabia	30–49	22.11%	26.90%	51.50%	31.90%	GAD-7	N.A.	277 (55.2%)	258 (51.4%)	N.A.
									PHQ-9				
Azoulay et al. (2020b)	1,001	20%	-	39–53	N.A.	N.A.	N.A.	34%	HADS	≥7	302 (30.2%)	465 (46.5%)	N.A.
Civantos et al. (2020a)	163	N.A.	Brazil	-	100%	-	-	25.80%	GAD-7	≥4	74 (45.5%)	26 (16.0%)	N.A.
									PHQ-9	≥3			
Civantos et al. (2020b)	349	N.A.	-	-	100%	-	-	39.30%	GAD-7	≥4	37 (10.6%)	167 (47.9%)	N.A.
									PHQ-9	≥3			
Luceno-Moreno et al. (2020)	1,422	N.A.	Spanish	43.88 ± 10.82	N.A.	N.A.	N.A.	86.40%	HADS	≥7	730 (51.3%)	1,128 (79.3%)	N.A.
Prasad et al. (2020)	347	N.A.	America	26–40	-	71.50%	28.50%	90.80%	GAD-2	≥4	79 (22.8%)	241 (69.5%)	N.A.
									PHQ-2	≥3			
Wang et al. (2020)	1,045	99.60%	China	-	14.30%	74%	11.70%	85.80%	HADS	≥7	142 (13.6%)	209 (20%)	N.A.
									ISI	≥8			
Xiong et al. (2020)	231	61.80%	China	-	-	100%	-	97.30%	GAD-7	≥4	61 (26.4%)	94 (40.8%)	N.A.
									PHQ-9	≥5			

*All studies are cross-sectional; the absolute number of patients for each category is included in the brackets; N.A., not available*.

**Table 2:** Modified Newcastle-Ottawa quality assessment scale and total score of each study.

**References**	**Modified Newcastle-Ottawa quality assessment scale**					**Score**
	**1**	**2**	**3**	**4**	**5**	
Hosseinzadeh-Shanjani et al. (2020)	^*^	-	^*^	-	^*^	3
Liu et al. (2020)	^*^	-	^*^	^*^	-	3
Guo et al. (2020)	^*^	^*^	-	^*^	^*^	4
Tan et al. (2020)	^*^	-	^*^	^*^	^*^	4
Wang et al. (2020)	^*^	^*^	^*^	^*^	-	4
An et al. (2020)	^*^	^*^	^*^	^*^	-	4
Azoulay et al. (2020a)	^*^	^*^	-	^*^	-	3
Chen X. et al. (2020)	^*^	-	-	^*^	^*^	3
Cheng et al. (2020)	^*^	-	-	^*^	^*^	3
Elhadi et al. (2020)	^*^	^*^	^*^	^*^	^*^	5
Giusti et al. (2020)	^*^	-	-	^*^	^*^	3
Hu et al. (2020)	^*^	^*^	-	^*^	^*^	4
Huang et al. (2020a)	^*^	^*^	^*^	^*^	^*^	5
Huang et al. (2020a)	^*^	-	^*^	^*^	-	3
Huang et al. (2020b)	^*^	-	^*^	^*^	^*^	4
Labrague and De Los Santos (2020)	^*^	-	-	^*^	^*^	3
Lai et al. (2020)	^*^	^*^	-	^*^	^*^	4
Li et al. (2020a)	^*^	^*^	^*^	^*^	^*^	5
Li et al. (2020b)	^*^	-	-	^*^	^*^	3
Li et al. (2020c)	^*^	^*^	-	^*^	^*^	4
Liu et al. (2020)	^*^	^*^	-	-	^*^	3
Lu et al. (2020)	^*^	^*^	-	^*^	-	3
Naser et al. (2020)	^*^	^*^	-	^*^	^*^	4
Ni et al. (2020)	^*^	-	^*^	^*^	^*^	4
Pouralizadeh et al. (2020)	^*^	-	-	^*^	^*^	3
Que et al. (2020)	^*^	^*^	-	^*^	^*^	4
Rossi et al. (2020)	^*^	^*^	-	^*^	^*^	4
Sandesh et al. (2020)	^*^	-	-	-	^*^	2
Si et al. (2020)	^*^	^*^	^*^	-	^*^	4
Song et al. (2020)	^*^	^*^	-	^*^	^*^	4
Tu et al. (2020)	^*^	-	-	^*^	^*^	3
Wańkowicz et al. (2020)	^*^	-	-	^*^	^*^	3
Xiao et al. (2020)	^*^	^*^	^*^	^*^	^*^	5
Xu et al. (2020)	^*^	^*^	-	^*^	^*^	4
Zhang et al. (2020a)	^*^	^*^	-	^*^	^*^	4
Zhang et al. (2020b)	^*^	^*^	-	^*^	^*^	4
Zhao et al. (2020)	^*^	^*^	-	-	^*^	3
Zhu et al. (2020a)	^*^	-	-	^*^	^*^	3
Zhu et al. (2020b)	^*^	^*^	^*^	^*^	^*^	5
AlAteeq et al. (2020)	^*^	-	-	-	-	1
Azoulay et al. (2020b)	^*^	^*^	-	^*^	-	3
Civantos et al. (2020a)	^*^	-	-	^*^	^*^	3
Civantos et al. (2020b)	^*^	-	-	^*^	^*^	3
Luceno-Moreno et al. (2020)	^*^	^*^	-	^*^	^*^	4
Prasad et al. (2020)	^*^	-	-	^*^	^*^	3
Wang et al. (2020)	^*^	^*^	^*^	^*^	^*^	5
Xiong et al. (2020)	^*^	-	-	^*^	-	2

*1. Representativeness of sample (The number of HCWs ≥ 65% of total sample); 2. Sample size > 600 HCWs; 3. Response rate > 80%; 4. The study employed validate measurement tools with appropriate cut-offs; 5. Adequate statistics and no need for further calculations.*

* *The study met the scoring criteria. -The study did not meet the grading criteria*.

### Prevalence of Anxiety

**Figure 2:**
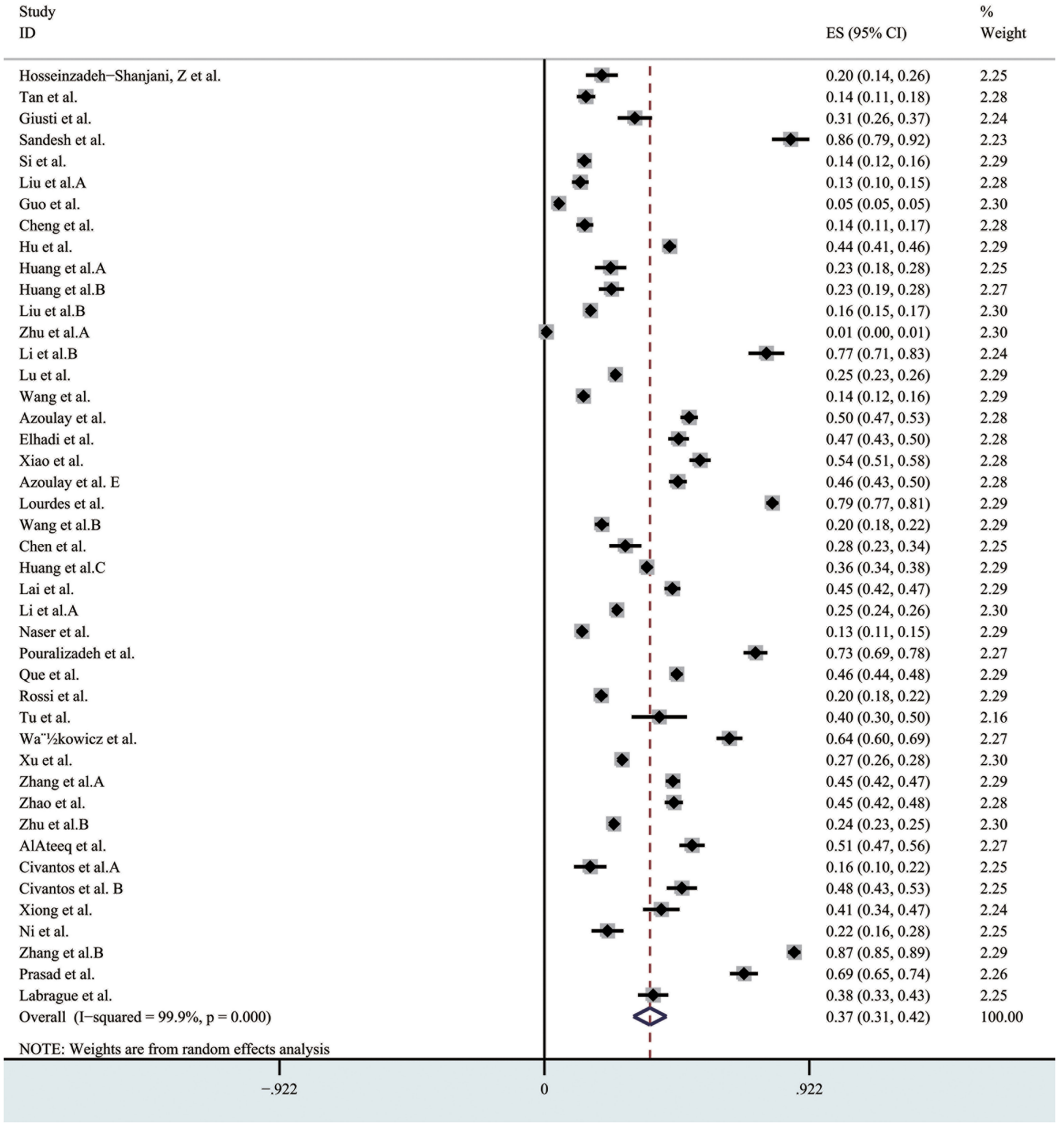
The pooled prevalence of anxiety. ES, effect size; CI, confidence interval; Weight, weight of each included study (degree of impact on pooled results), The larger the weight is, the greater the influence on the combination result is.

A total of 44 studies reported the prevalence of anxiety, and the pooled prevalence of the anxiety was 37% (95% CI 0.31–0.42, I^2^ = 99.9%) as shown in Figure 2. In the sensitivity analysis, no study affected the pooled prevalence by over 3% when excluded. As far as the assessment tool, studies using different validated scales included: Depression, Anxiety, Stress Scale (DASS); Self-Rating Anxiety Scale (SAS); Hamilton Anxiety Scale (HAMA); Hospital Anxiety and Depression Scale (HADS); The 7-item Generalized Anxiety Disorder scale (GAD-7); Generalized Anxiety Disorder-2 (GAD-2) and The COVID-19 Anxiety scale.

### Prevalence of Depression

**Figure 3:**
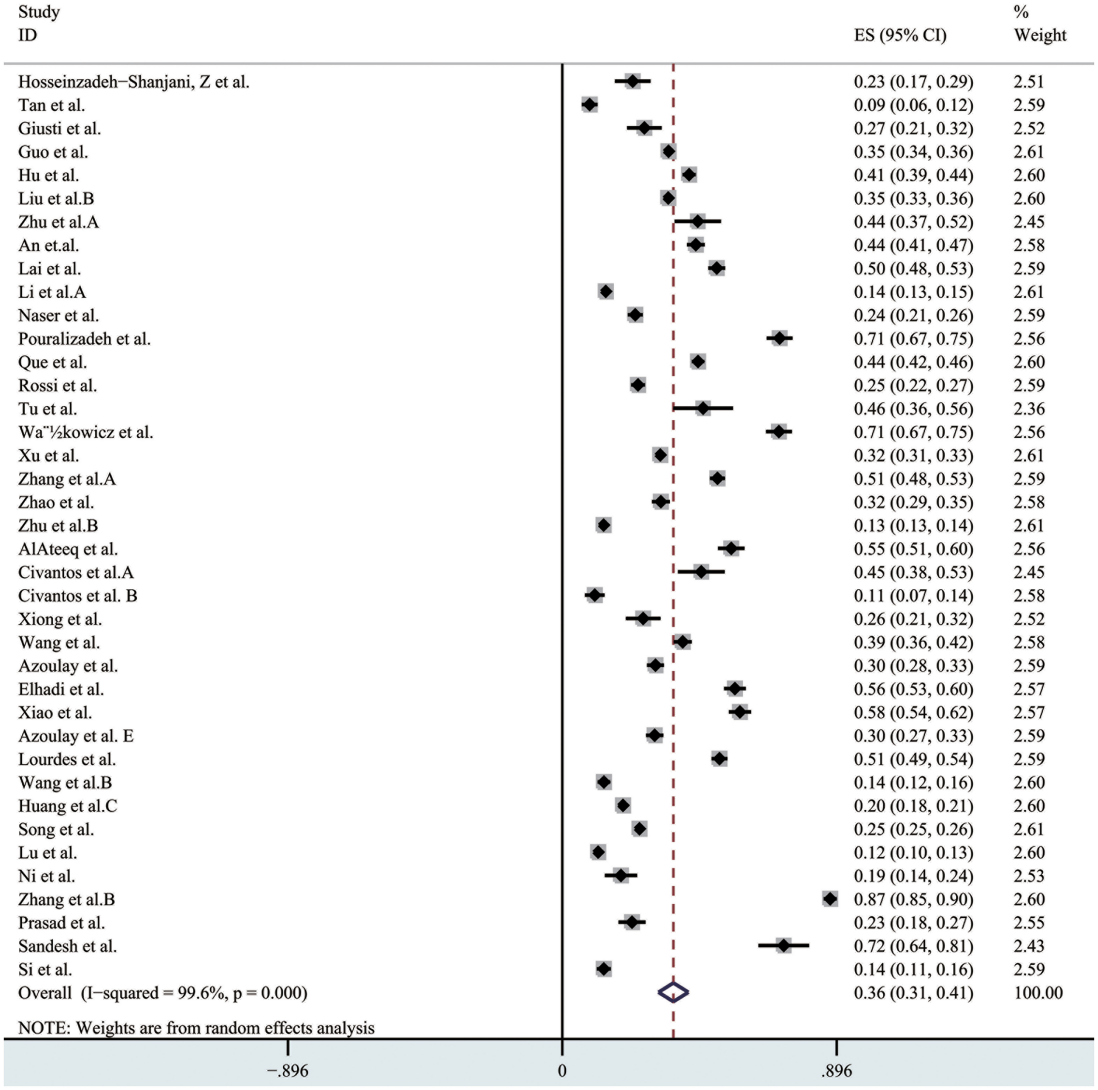
The pooled prevalence of depression. ES, effect size; CI, confidence interval; Weight, weight of each included study (degree of impact on pooled results), The larger the weight is, the greater the influence on the combination result is.

Depression were estimated in 39 studies, and the pooled prevalence of the depression was 36% (95% CI 0.31–0.41, I^2^ = 99.6%), as presented in Figure 3. In the sensitivity analysis, no study affected the pooled prevalence by over 1% when excluded. Different validated scales used to measure depression included: Depression, Anxiety, Stress Scale (DASS); Self-Rating Depression Scale (SDS); the 9-item Patient Health Questionnaire (PHQ-9); The Hospital Anxiety and Depression Scale (HADS); The Center for Epidemiologic Studies Depression Scale (CES-D); Hamilton Depression Scale (HAMD) and Patient Health Questionnaire-2 (PHQ-2).

### Prevalence of Insomnia

**Figure 4:**
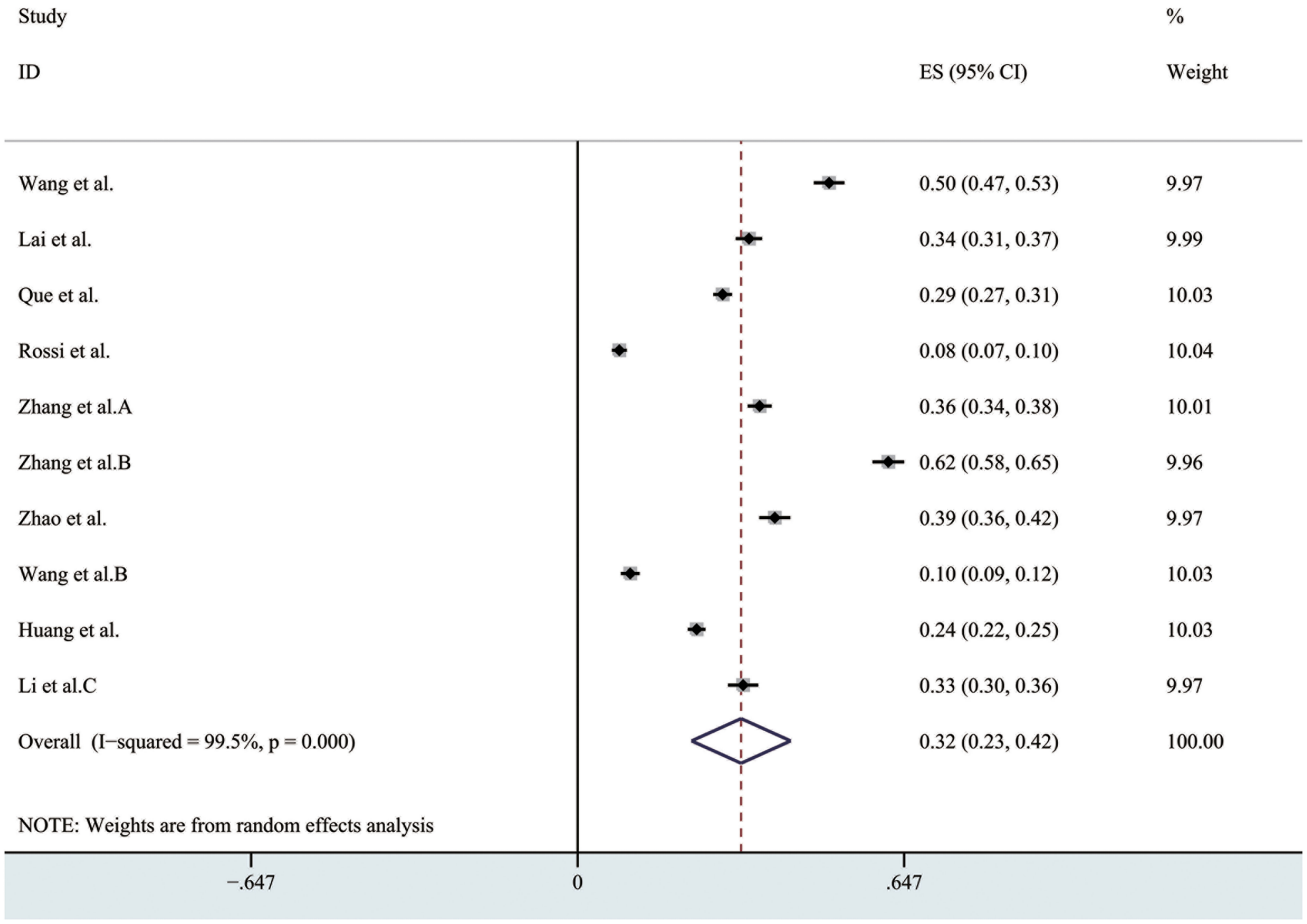
The pooled prevalence of insomnia. ES, effect size; CI, confidence interval; Weight, weight of each included study (degree of impact on pooled results), The larger the weight is, the greater the influence on the combination result is.

There were 10 studies which reported the prevalence of insomnia, and the overall prevalence of the insomnia was 32% (95% CI 0.23–0.42, I^2^ = 99.5%) as presented in Figure 4. In the sensitivity analysis, no study affected the pooled prevalence by over 3% when excluded. The evaluation tool using in these studies included: the seven-item Insomnia Severity Index (ISI); PSQI (Pittsburgh Sleep Quality Index) scale and Athens Insomnia Scale (AIS).

### Subgroup Analysis

**Figure 5:**
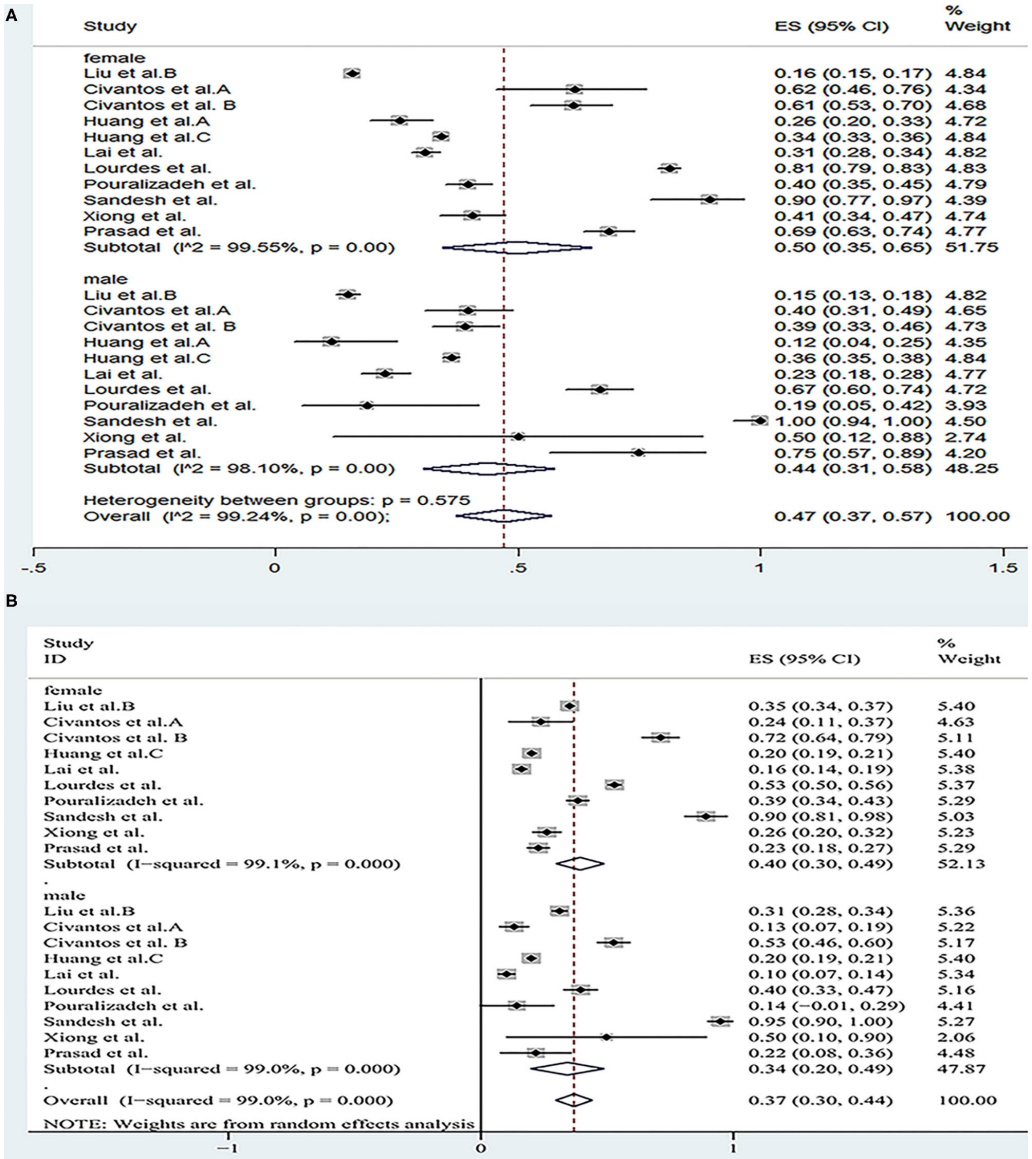
The forest map based on the prevalence of anxiety and depression among healthcare workers of different genders. **(A)**: A forest map based on the incidence of anxiety among health care workers of different genders; **(B)**: A forest map based on the incidence of depression among health care workers of different genders. ES, effect size; CI, confidence interval; Weight: weight of each included study (degree of impact on pooled results), The larger the weight is, the greater the influence on the combination result is.

**Figure 6:**
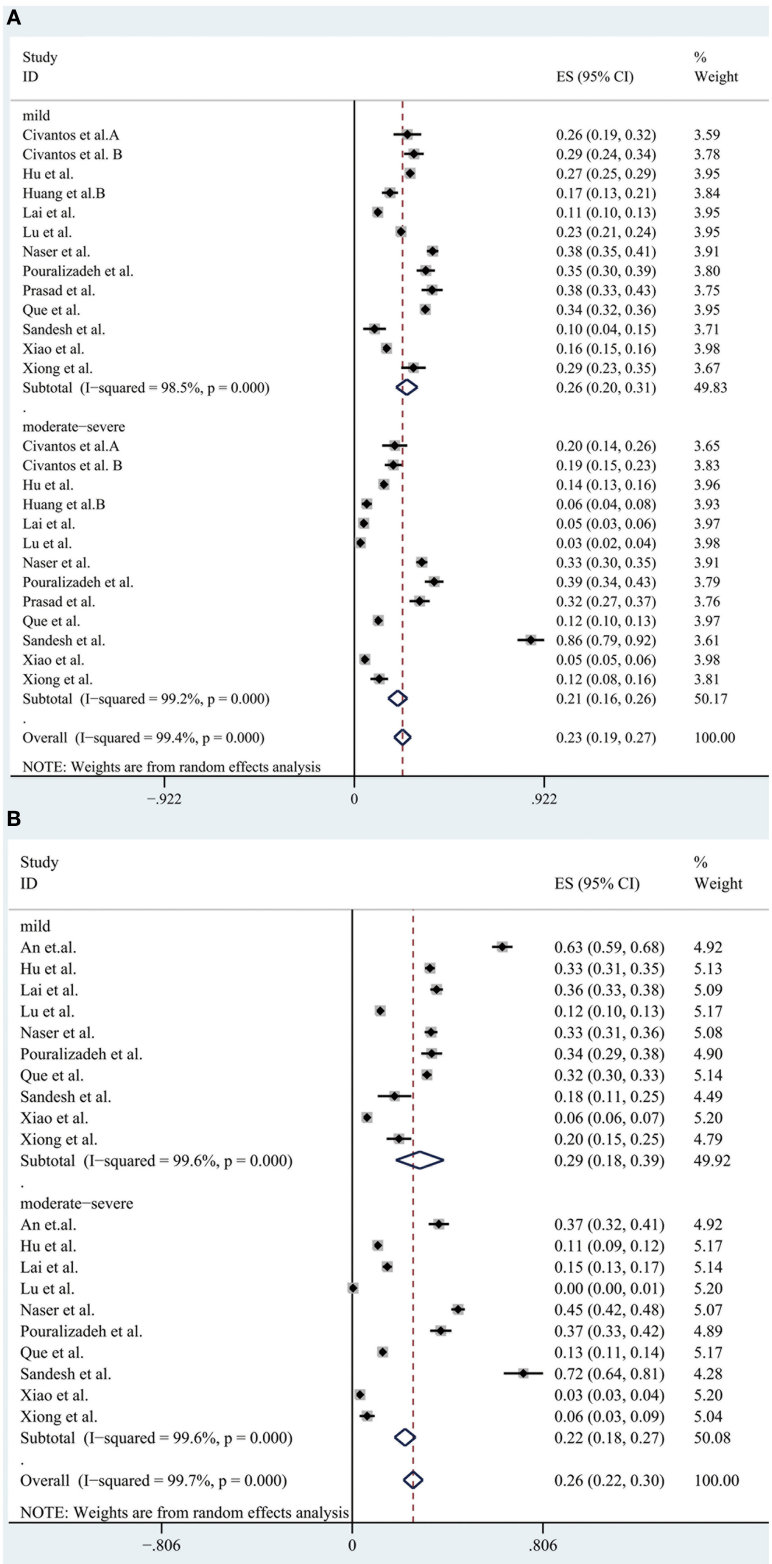
A forest map based on the incidence of various levels of anxiety and depression among medical personnel. **(A)**: A forest map based on the incidence of various levels of anxiety among medical personnel; **(B)**: A forest map based on the incidence of various levels of depression among medical personnel. ES, effect size; CI, confidence interval; Weight: weight of each included study (degree of impact on pooled results), The larger the weight is, the greater the influence on the combination result is.

**Figure 7:**
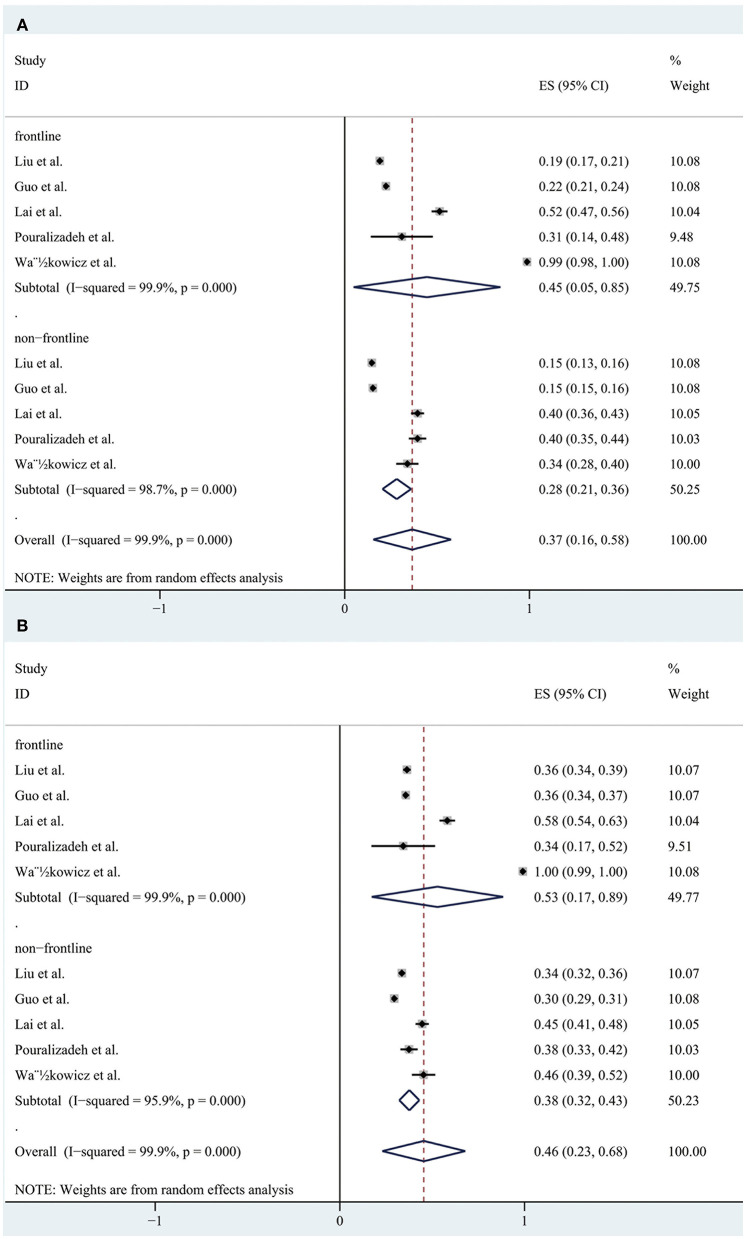
A forest map based on the prevalence of anxiety and depression between front-line and non-frontline medical staffs. **(A)**: A forest map based on the incidence of anxiety between front-line and non-frontline medical personnel; **(B)**: A forest map based on the incidence of depression between front-line and non-frontline healthcare workers. ES, effect size; CI, confidence interval; Weight: weight of each included study (degree of impact on pooled results), The larger the weight is, the greater the influence on the combination result is.

**Figure 8:**
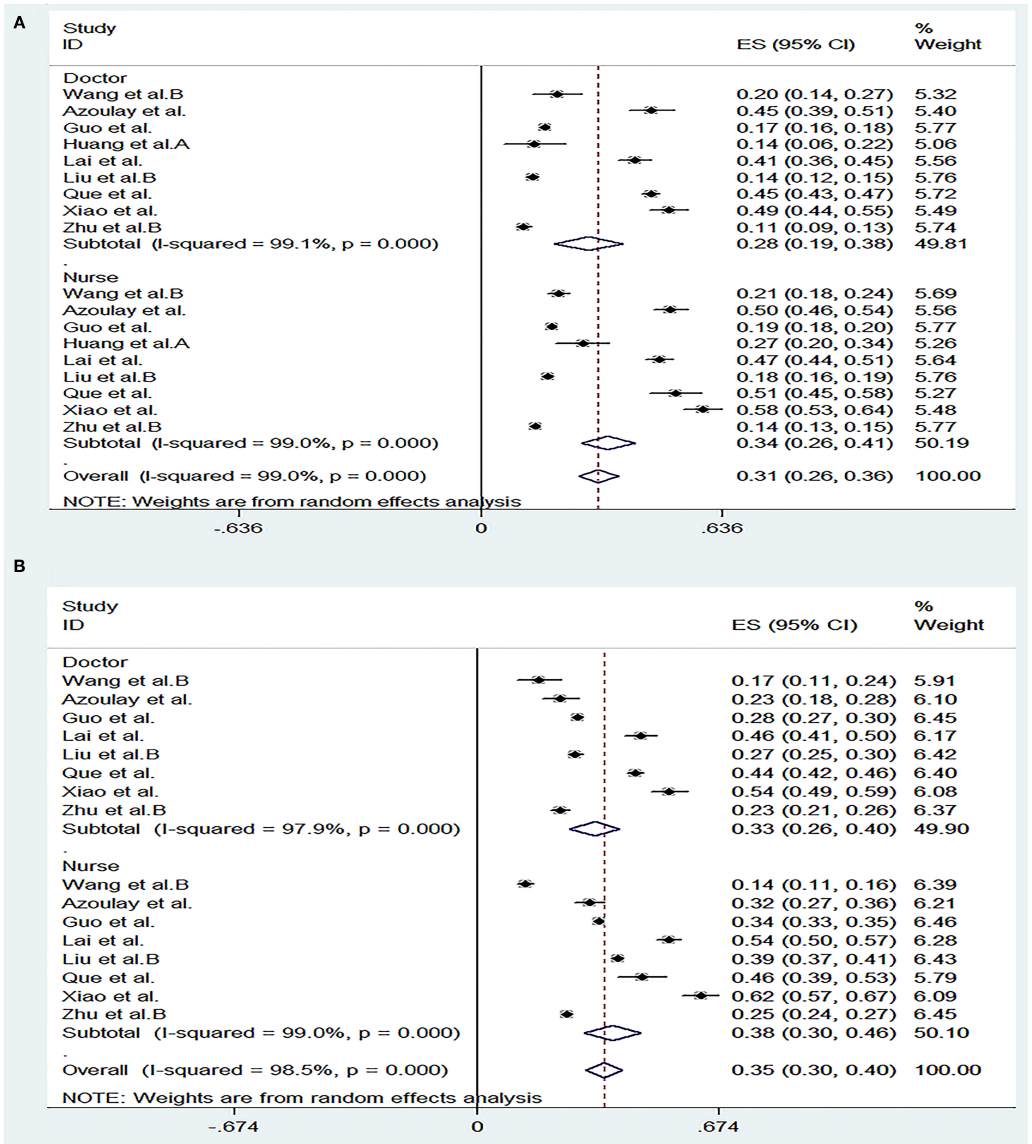
A forest map based on the prevalence of anxiety and depression between doctors and nurses. **(A)**: A forest map based on the incidence of anxiety between doctors and nurses; **(B)**: A forest map based on the incidence of depression between doctors and nurses. ES, effect size; CI, confidence interval; Weight, weight of each included study (degree of impact on pooled results), The larger the weight is, the greater the influence on the combination result is.

We conducted a subgroup analysis of the morbidity of the anxiety and depression by gender, severity, risk and profession. The results are shown in Figures 5–8, respectively.

A total of 11 studies reported the prevalence of anxiety by gender and the pooled prevalence was 50, 36% for female and male, respectively. The severity of the anxiety was divided into two groups including “mild” group and “moderate-severe” group and data could be obtained in 13 articles. The overall prevalence of anxiety for “mild” group was 26 and 21% for “moderate-severe” group. In the frontline and non-frontline groups, data on prevalence of anxiety were available in 5 literatures, with respective values of 45 and 28%. In addition, nine literatures (Al Sulais et al., 2020; Azoulay et al., 2020a; Guo et al., 2020; Huang et al., 2020a; Lai et al., 2020; Liu et al., 2020; Que et al., 2020; Xiao et al., 2020; Zhu et al., 2020b) reported the prevalence of anxiety of doctors and nurses. The overall prevalence of anxiety for “doctor” group was 28%, and 34% for “nurse” group. For depression, data could be extracted from 10 articles according to gender, with a pooled prevalence of 40% for female and 34% for male. As far as the severity of depression, there were also 10 studies reported the morbidity of depression, with values of 29% for “mild” group and 22% for “moderate-severe” group respectively. Regarding to the “frontline” group and “non-frontline” group, the prevalence of depression was 53% in the former and 38% in the later which were calculated from the same five articles as anxiety. The prevalence of depression could be detected in eight literatures, with a pooled prevalence of 33% for doctors and 38% for nurses. As far as insomnia, a subgroup analysis was not performed due to the limited available data.

## Discussion

Results of a systemic review and meta-analysis of 47 studies indicated that a large proportion of healthcare workers suffered from the adverse psychological impact of COVID-19. Symptoms such as anxiety, depression and insomnia were analyzed as indicators of psychological effects of healthcare staffs during the COVID-19 epidemic, with respective values of 37% (31–42%), 36% (31–41%), and 32% (23–42%). The results of subgroup analysis showed that the incidence of anxiety and depression was significantly increased in females. After statistical analysis and calculation of the included data, the proportion of the “mild” group was higher than that of the “moderate-severe” group. The prevalence of anxiety and depression of frontline HCWs was much higher than non-frontline HCWs. Furthermore, nurses had higher rates of anxiety and depression than doctors.

Since COVID-19 is novel and has never been explored, and its horrible infectivity and mortality generate great stress throughout healthcare staffs especially on the frontline healthcare professionals, which become a major factor causing anxiety depression and insomnia among HCWs (Salari et al., 2020). In addition, there are also other factors, for example, insufficient medical protective equipment, contact ban with relatives, transfer to another ward, work overload and so on (Sandesh et al., 2020; Shigemura et al., 2020; Wańkowicz et al., 2020) that would lead to mental health problems for medical staff. In view of the above, several practical steps should be implemented by government officers and hospital administrators in order to prevent the occurrence of mental illness and relieve the mental and physical stress of the healthcare staffs, for example, adequate supplies of protective equipment (Koh et al., 2005; Chen et al., 2006; Kim and Choi, 2016), appropriate work shift with regular sleep (Ho et al., 2005; Lee et al., 2005; Kang et al., 2018), clear communication (Bai et al., 2004; Goulia et al., 2010; Kang et al., 2018), and video contact with their families and friends (Azoulay et al., 2020a; Kisely et al., 2020). Furthermore, it's extremely necessary to provide timely and professionally tailored mental health support through media or multidisciplinary teams (Pappa et al., 2020). Whether the medical protective equipment provided by the government and society is sufficient, whether the social support is enhanced, etc., all of these factors, to a certain extent, can enhance the mental strength of medical staff and reduce the incidence of mental illness. We believe it is necessary to conduct this meta-analysis based on the research published so far to address the psychological condition of the HCWs.

Compared with the recent meta-analysis concerning the psychological impacts of COVID-19 among healthcare professionals which included 13 articles and reported the prevalence of anxiety, depression and insomnia were 23.2, 22.8, and 34.2% respectively, our study found a similar prevalence of insomnia among healthcare staffs but the prevalence of anxiety 37% and depression 36% were much higher than the prior study. We consider that as the epidemic worsens and the number of cases increase rapidly all over the world, the mental and physical stress faced by medical staff in each country is also increasing.

According to the result of the subgroup analysis, the incidence of anxiety and depression among female medical staffs was higher than that of male. The results of epidemiological studies showed that women were at a higher risk of depression (Lim et al., 2018). There are many reasons for this gap between men and women. For example, genetic factors might play a part, but empirical evidence for their potential to explain the gender gap in depression is still scarce (Albert, 2015; Kuehner, 2017). In addition, a ruminative response style, that is, the tendency to passively and repetitively analysis one's distress, problems, and concerns, without taking actions, has been proposed to account for a substantial part of the gender gap in depression. Two meta-analyses identified higher rumination tendencies in women than in men (Kuehner, 2017). However, of the studies included in our subgroup analysis, most had a higher percentage of female responders than male, and only four (Civantos et al., 2020a,b; Lai et al., 2020; Sandesh et al., 2020) (25.8, 39.3, 23.3, 42.9%, respectively) had a lower percentage of women than men. This selection bias may exist. According to the statistical results of the literature, we included that the prevalence rate of anxiety and depression appeared to be higher in “mild” group, while moderate and severe symptoms were less common among the participants. In our opinion, this result suggests that we ought to detect and intervene the mental health status of medical staffs timely and efficiently in order to prevent the occurrence of adverse mental illness, thus effectively reducing the incidence of anxiety and depression. Furthermore, we also find that a great proportion of the frontline healthcare workers suffered from anxiety and depression, since frontline healthcare professionals treating patients with COVID-19 are likely to be exposed to the highest risk to be infected because of their close, frequent contact with patients and longer hours than usual. In addition, these people are exposed to emotionally challenging interactions with the sick and critically ill patients and they tend to pay more attention to their own and their families' health. Moreover, they are subject to occupational overload due to staff shortages and insufficient personal protective equipment (Rossi et al., 2020; Wańkowicz et al., 2020). This highlighted the significance to take more effective and precautionary measures to protect frontline healthcare workers from the psychological damage at the governmental level and the personnel level (Luo et al., 2020). The higher overall prevalence of anxiety and depression is observed in nurses. There are several reasons to consider this. Firstly, nurses are relatively young and mostly female which may be an important reason. In addition, nurses are responsible for the collection of sputum for virus detection, which is the most dangerous work (Guo et al., 2020), and they need to act as “gatekeepers” responsible for educating and monitoring the practices of staff and visitors and also had an increased workload as they took on duties of other staff (Mitchell et al., 2002). All of these may contribute to the higher incidence of anxiety among nurses than doctors.

Overall, the findings of the current study may have some clinical implications. First, the COVID-19 pandemic situation has been effectively controlled in some countries, but the situation in other countries is still not optimistic. We must take adequate measures to prevent possible the next outbreak of the epidemic. We clearly confirm that HCWs who under high physical and mental health burden are still at a higher risk of suffering from psychological disease. It is urgently to provide psychological assistance to medical personnel who could help the governments better prepare for future outbreaks of unexpected infectious diseases. Second, our study showed that frontline HCWs presented higher prevalence of anxiety and depression. In fact, they did not want their families and friends worry about them and were afraid of bringing the virus to their home. In addition, patients' not cooperation with medical measures, unable to deal with patients' anxiety, panic, and other emotional problems, shortage of protective equipment were the main factors resulting psychological stress (Chen Q. et al., 2020). Therefore, it is not only necessary to ensure adequate equipment supply and timely psychological support for medical staffs, but also important to provide psychological support for patients, as it indirectly affects the mental and psychological state of frontline HCWs. Also, to a certain extent, the workload and difficulty of frontline medical work will be reduced. We held the opinion that a special research can be conducted to study which factors (little is known about the new virus/disease, limited resource, long work hours, contact ban with relatives, work overload, etc.) have a serious psychological impact on medical staff in the new epidemic, and what measures can be taken to effectively reduce the psychological pressure on medical staff in the future study. Third, the second wave of the outbreak in many countries has been kicked off, due to the outbreak of the first wave made a great influence on the spirit of the medical personnel psychology, therefore, we think it is necessary for medical workers, especially women and healthcare workers who have fought on the front line to carry out a psychological evaluation and to give professional guidance in time, in order to prevent the second pandemic from having a more severe impact on them.

There are several strengths and limitations to our review. Compared to the last systematic review and meta-analysis that comprised 13 studies from Asian countries (*n* = 33,062), the current meta-analysis included more studies (47 studies from 12 countries) with a much bigger sample size (*n* = 81,277). Psychological conditions of frontline and non-frontline HCWs were further investigated, which has some guiding implications for medical personnel to provide psychological assistance when facing different risks. The inherent heterogeneity across studies is also one of the main considerations for the limitations on research. While all of these studies reported prevalence of anxiety, depression or insomnia, several of them used the same test, but screened the population using different assessment scales and set different thresholds which may have an effect on the outcome. In addition, only three symptoms were meta-analyzed in this article due to the lack of relevant studies on other psychological symptoms of healthcare workers. Therefore, related studies can be conducted on other psychological effects of the COVID-19 on medical staff in the future. Moreover, most of the included studies are from China, data from other countries are still a few. At the present stage, the severity of the epidemic varies from country to country; the impact on the mental health of medical workers also varies greatly. In addition to the study in China, 16 of the included studies were conducted in 13 different countries. It is not reliable to describe the psychological status of medical staff in each country according to the limited literature collected so far. Therefore, if more literatures are collected in the future, further analysis can be made in this aspect. All quantitative studies were cross-sectional surveys with a short follow-up duration. Psychological status of medical personnel will change as the epidemic progresses, so it is not possible to extrapolate from these studies only the long-term mental health effects and how the basic rates of these mental health symptoms relate to other periods. Some literatures reported age (MD ± SD) or age range of the study group, but some did not report (age not applicable, Table 1), so we could not analyze age as a factor. Although most of the study subjects were between the ages of 25 and 45 (young adults), we still can't rule out the possibility that age may affect the results. It is also a limitation of our study.

## Conclusions

The COVID-19 pandemic has created an emergency state and caused heavy psychological impact among HCWs. The prevalence of anxiety and depression are significantly higher in female HCWs than males, also in the frontline HCWs than non-frontline HCWs. In addition to quickly establish programs that provide knowledge on the virus, timely psychological counseling and intervention ought to be implemented for HCWs in order to alleviate their anxiety and improve their general mental health.

## Data Availability Statement

The original contributions presented in the study are included in the article/supplementary material, further inquiries can be directed to the corresponding authors.

## Author Contributions

LY: funding acquisition, conceptualization, and methodology. LC: supervision, review, and editing. MW, TS, YW, and JL: investigation and data curation. PS: data curation, original draft preparation, writing, and editing. All authors contributed, reviewed, and approved the final manuscript.

## Conflict of Interest

The authors declare that the research was conducted in the absence of any commercial or financial relationships that could be construed as a potential conflict of interest.
